# Post-treatment time to symptom resolution and associated factors in a cohort of Ugandan men with urethral discharge syndrome

**DOI:** 10.1186/s12879-025-11196-8

**Published:** 2025-06-08

**Authors:** Courtney Pasco, Yisi Liu, Junyi Zhou, Ethan Gough, Annet Onzia, Rosalind Parkes-Ratanshi, Johan H. Melendez, Peter Kyambadde, Yukari C. Manabe, Matthew M. Hamill

**Affiliations:** 1 Johns Hopkins University School of Medicine, Baltimore, MD USA; 2 Bloomberg School of Public Health, Johns Hopkins University, Baltimore, MD USA; 3 Infectious Diseases Institute, Kampala, Uganda; 4 University of Cambridge, Cambridge, UK; 5 Division of Infectious Diseases, Johns Hopkins University School of Medicine, 5200 Eastern Avenue, Baltimore, MD 22114 USA; 6 Ministry of Health, STD/AIDS Control Programme, Kampala, Uganda

**Keywords:** Uganda, Men, Urethritis, Urethral discharge syndrome, Symptom resolution

## Abstract

**Introduction:**

Ugandan men with urethral discharge syndrome (UDS) have high burdens of curable sexually transmitted infections (STIs) and HIV. STI complaints are treated syndromically, but post-treatment symptom resolution (SR) data are lacking in this group. This study estimated the time from treatment to symptom resolution (TTR) and examined associations with sociodemographic and behavioral factors and TTR.

**Methods:**

250 men with UDS were recruited at health centers in Kampala, Uganda. Participants underwent point-of-care testing for HIV/syphilis, and urogenital samples were retrospectively analyzed for *Chlamydia trachomatis* (CT), *Neisseria gonorrhoeae* (NG), *Mycoplamsa genitalium* (MG), and *Trichomonas vaginalis* (TV) using Aptima nucleic acid amplification tests (Hologic Inc., Marlborough, MA, USA). Socio-behavioral data were collected by questionnaire. Participants received follow-up calls at 14-, and 21-days post-enrollment to assess SR, antibiotic adherence, and sexual behaviors. Differences between participants by SR at day 14 were determined by Fisher Exact test, Wilcoxon rank-sum test, Chi-squared test, and Welch’s t-test as appropriate. Univariable and multivariable accelerated failure time (AFT) models were used to identify associations between participant factors and TTR.

**Results:**

Of 239 (95.6%) participants who completed day 14 follow-up surveys, 37 (16%) did not have SR by 14-days post-enrollment and treatment initiation. Median (IQR) TTR was 4.0 (3.0,6.0) days. Delayed TTR was associated with previous episodes of UDS in the prior six months (2.0 vs. 1.4, *p* = 0.010) and negative tests for CT/NG/MG/TV (35% vs. 15%, *p* = 0.004). These relationships held true when controlling for potential confounders including prior antimicrobial use, possible reinfection following sexual exposures post-enrollment, treatment non-adherence, HIV status, and other behaviors associated with increased vulnerabilities to STIs.

**Conclusion:**

Delayed TTR was associated with prior UDS episodes. Negative tests for common curable STIs were associated with delayed TTR suggesting the possible role of other infectious or non-infectious etiologies. The underlying mechanisms of delayed SR, e.g., reinfection, treatment failure, or dysregulated mucosal immunity, warrant further exploration.

**Supplementary Information:**

The online version contains supplementary material available at 10.1186/s12879-025-11196-8.

## Introduction

Sub-Saharan Africa bears the highest burden of curable sexually transmitted infections (STIs) and HIV [1]. The limited diagnostic capacity for non-HIV STIs results in syndromic management including for urethral discharge syndrome (UDS), where a broad range of antimicrobials are used to cover the most common STIs [2]. STIs increase transmission of and susceptibility to HIV by raising HIV viral load in semen and causing mucosal inflammation, in the absence of suppressive antiretroviral therapy (ART) or effective pre-exposure prophylaxis [3]. Persistent STI symptoms may indicate inappropriate treatment, antimicrobial-resistance (AMR), or infection with organisms like herpes simplex virus (HSV) or *H. influenzae* [4] not routinely tested for or treated in African settings.

STI transmission is influenced by the length of time an individual remains infected. Persistent symptoms may indicate that the underlying infection is still present and presumably has the capacity for onward transmission [4–6]. Thus, US Centers for Disease Control and Prevention (CDC) Guidelines recommend abstention from sex after STI treatment until treatment completion, partner treatment, and symptom resolution [4]. The British Association for Sexual Health and HIV recommend abstention from sex until 7 days after both patient and partners have completed treatment for gonorrhoea [7]. Infectious disease symptoms, including those caused by STIs, are typically due to a combination of pathogen factors and host responses. Male urethral gonococcal infection is associated with increased inflammatory cytokines [8], and symptomatic infection, specifically, is associated with an increase in urinary IL-1β, thought to be produced by infiltrating leukocytes [8]. Male urethral *Neisseria gonorrhoeae* (NG) is typically symptomatic [9]. In penile urethral infections, particularly when due to NG, symptoms may serve as an important, easily measured surrogate marker for ongoing infection.

Much of the existing data on time to resolution (TTR) of symptoms is based on experimental gonococcal infections and treatments [10, 11]. Previous studies showed NG was no longer detected on cultures from urine, mucosa, and semen specimens within 24 h after single-dose treatment in a male cohort [10]; a negative RNA assay in a median of 2 days in a cohort of both men and women [11], and symptom resolution in the same time frame in a male cohort [12]. In a study of men who have sex with men with non-gonococcal urethritis, the median time to all-cause symptom resolution after azithromycin therapy was 7 (95%CI 5–9) days, 37% had symptoms for more than 7 days post-treatment [13]. For those with NGU due to CT-, MG-, and non-CT/MG-NGU, TTR was 4 (95%CI 2–6) days [13]. In Africa, there is an absence of data on TTR in men with UDS, which is predominantly caused by NG [14].

The objectives of this analysis were twofold; first to estimate time from treatment-initiation to UDS symptom resolution and second, to examine associations between sociodemographic and behavioral factors and TTR. Additionally, we sought to better understand how specific STIs, STI coinfections, and antibiotic prescribing practices were associated with UDS symptom resolution.

## Materials and methods

Full details of the study design, recruitment strategy, and cross-sectional data have been published elsewhere [14]. Briefly, the World Health Organization’s Enhanced Gonococcal Antimicrobial Surveillance Program (EGASP) was initiated in Uganda in 2016 to assess resistance trends in gonorrhoea [15]. Between October 2019 and November 2020, a total of 250 men with self-reported symptoms of UDS, including dysuria and penile discharge, were recruited consecutively at six of 10 EGASP-participating government health centers in Kampala, Uganda. Participants received standard of care (SOC) and clinical evaluation before study enrollment. Local SOC included syndromic management of UDS comprising single dose oral cefixime 400 mg and doxycycline 100 mg twice daily for 7 days [16], advise on consistent condom use, and encouragement to bring partners in for treatment. Participants did not take antimicrobial therapy until after sample collection.

After study enrollment, participants underwent onsite testing for HIV and syphilis antibodies using rapid tests as previously described [14, 17]. After transportation to the Johns Hopkins University International STD laboratory, penile-meatal swabs were tested using nucleic acid amplification tests (NAAT) for NG, *Chlamydia trachomatis* (CT), and *Trichomonas vaginalis* (TV). Both penile-meatal swabs and urine samples were tested using NAAT for *Mycoplasma genitalium* (MG) (all NAATs: Hologic Inc., Marlborough, MA, USA). Demographic details, sexual and social behaviors, including self-reported alcohol use, engagement in transactional sex, and condom use were assessed in a structured interview by trained research staff as previously reported [14] (supplementary material). Participants received transport reimbursement of Ugandan shillings 20,000 (approximately USD 5–6) for their enrollment visit only.

After completion of the enrollment visit, participants received follow-up telephone calls at 14-, and 21-days. Again, a structured interview was conducted by trained research staff to assess receipt of treatment, self-reported symptom resolution, antibiotic adherence, and sexual and other behaviors, including engagement in sex since diagnosis, and disclosure of UDS diagnosis to partners. Symptom resolution was assessed as both a binary variable by asking participants whether their symptoms had resolved at the time of follow-up, and continuous variable by asking participants how many days it took for their symptoms to resolve post-treatment.

### Ethical approval and consent to participate

In Uganda, the Joint Clinical Research Center at Makerere University (JC0919), and the Ugandan National Council for Science and Technology (HS455ES) approved the original study. Additionally, the Johns Hopkins Institutional Review Board (IRB00215298) approved the study protocol. No study procedures were commenced until written informed consent had been obtained in either Luganda or English. All study procedures were performed in accordance with the relevant guidelines and regulations of the Declaration of Helsinki.

### Statistical analysis

Sociodemographic, clinical, and behavioral characteristics were described for the total study sample and by symptom resolution status at follow-up day 14 using frequencies and proportions or averages and standard deviations (SD) for categorical or numeric data, respectively. Previous studies [18, 19] have shown that symptom resolution from initial treatment for urethral infections was best assessed at follow-up day 14 for the following reasons: (i) most tests for infections and genital symptoms would have resolved within 14 days [18, 19], the US CDC suggest that if STI symptoms persist a few days after completion of treatment for chlamydia that the individual seek medical attention [4]; and (ii) the risk of reinfection and recall bias increases with time, making the 21-day time point less reliable to assess symptom resolution of the index UDS episode. Differences between study participants by symptom resolution at day 14 were determined by Fisher Exact test, Wilcoxon rank sum test, Chi-squared test, and Welch’s t-test as appropriate.

To identify factors associated with symptom resolution, we used Accelerated Failure Time (AFT) regression models for time-to-event data to estimate the relative change in time to symptom resolution in exposed versus unexposed participants (failure time ratio) for each risk factor investigated [20]. If the relative failure time ratio was > 1, then the analyzed risk factor lengthens time to resolution. Conversely, if the relative failure time ratio was < 1, then the risk factor shortens time to resolution. Participants who did not experience full symptom resolution by day 14 were right-censored. Differences between groups were visualized using Kaplan-Meier survival curves with *p*-values generated by log-rank test. Multivariable AFT regression models were fitted to estimate independent associations between risk factors and time to the symptom resolution. To minimize Table 2 bias [21], a separate multivariable model was fitted for each risk factor of interest, with covariates selected a priori based on subject matter knowledge and existing literature. AFT models were fitted using Stata version 18 (College Station, Texas, USA).

## Results

### Participant characteristics, time to resolution of symptoms, and STI prevalence

Of the 250 participants, 239 (95.6%) completed telephone follow-up surveys on day 14; the median time between enrollment and follow-up call was 14 days (IQR = 0, range 14–18 days); 220 (88.0%) completed day 21 follow up. Overall, the median (IQR) time to symptom resolution was 4.0 (3.0,6.0) days; the range was 1–18 days; 37 (15.5%) did not have symptom resolution by day 14. STI prevalence using NAAT and HIV prevalence using the Ugandan rapid sequential testing algorithm are described in Table 1, which also describes TTR by individual urethral STI and sociobehavioral factors in participants with and without symptom resolution by day 14. Of those with symptom resolution, 31 of 202 (15.3%) had negative tests for CT/NG/TV/MG compared to 13 of 37 (35.1%) in those with delayed TTR. There was a non-significant association between MG positivity and incomplete resolution (*p* = 0.06); unexpectedly a history of engagement in transactional sex was associated with symptom resolution.

**Table 1:** Bivariable analysis with symptom resolution as a binary variable assessed at day 14

Variable	All Participants (*n* = 239)	Incomplete Resolution at day 14 (*n* = 37)	Complete Resolution at day 14 (*n* = 202)	*P* Value
Age (years)	28.1	30.9	27.6	0.08^1^
Positive HIV test	19.7%	35.1%	16.8%	**0.01** ^2^
Number of episodes of UDS*	1.5	2.0	1.4	**0.01** ^3^
Number of sexual partners*	3.0	4.2	2.7	0.86^3^
Alcohol use around sex*	41.4%	32.4%	43.1%	0.23^2^
Drug use around sex*	2.9%	5.4%	2.5%	0.33^2^
Never used condoms*	40.2%	43.2%	39.6%	0.68^2^
Engaged in transactional sex*	62.8%	43.2%	66.3%	**0.008** ^2^
Engaged in sex post-enrollment	30.5%	43.2%	28.2%	0.07^2^
Symptom duration ≥ 6 days prior to enrollment	58.2%	67.6%	56.4%	0.21^2^
Used antibiotics in 14 days prior to enrollment	40.2%	48.6%	38.6%	0.25^2^
STI results^β^				
Negative for NG, CT, TV, MG	18.4%	35.1%	15.3%	**0.004** ^2^
Positive for CT	20.5%	24.3%	19.8%	0.53^2^
Positive for NG	64.9%	27.0%	71.8%	**< 0.001** ^2^
Positive for TV	2.1%	2.7%	2.0%	0.53^4^
Positive for MG	12.1%	21.6%	10.4%	0.06^2^
Antibiotic use				
Received CT/NG antibiotic coverage^µ^	93.3%	91.9%	93.6%	0.71^2^
Received only CT/NG antibiotic coverage (SOC)^α^	26.4%	32.4%	25.2%	0.36^2^
Adhered to prescribed antibiotics	98.3%	91.9%	99.5%	**0.001** ^2^
Received additional treatment post-enrollment	10.9%	40.5%	5.4%	**< 0.001** ^2^

1 Calculated with Welch’s t-test

2 Calculated with Chi-squared test

3 Calculated with Wilcoxon rank-sum test

4 Calculated with Fisher Exact test

* By self-report in the 6 months prior to study enrollment; ^µ^Received cefixime and doxycycline, also received other antimicrobials; ^α^Received only cefixime and doxycycline

^β^ Time to symptom resolution (in days) by curable STI (median [min, max]). Number of TV cases were too small for meaningful analysis

1. CT: 4 [2,10]

2. NG: 4 [1,12]

3. MG: 5 [4,12]

4. ≥ 1 curable STI: 4 [1,12]

### Factors associated with time to symptom resolution

Similar relationships were demonstrated in the AFT model that was used to evaluate TTR as a continuous variable (Table 2). For example, TTR increased with a history of previous UDS; every additional episode of UDS reported in the six months prior to enrollment increased TTR by a factor of 1.36 (*p* < 0.001). TTR was also associated with negative tests for urethral STIs; testing negative for NG, CT, MG, and TV increased mean time to symptom resolution by a factor of 1.58 (*p* = 0.001). Age (expressed as a continuous variable) (1.016, *p* = 0.004), and a positive MG NAAT (1.399, *p* = 0.04), not significant in the bivariable analysis, were significantly associated with TTR in the AFT. Conversely, adherence to prescribed antibiotics, significant in the bivariable analysis, was no longer significant (0.467, *p* = 0.13). A positive NAAT for NG was associated with a shorter TTR in the bivariable and AFT analyses.

**Table 2:** Accelerated failure time regression models for time to symptom resolution

Variable	Failure Time ratio	Standard Error	95% CI^¥^	*P* value
Age (Categorical)	1.11	0.109	0.92–1.35	0.28
Age (Continuous)	1.02	0.006	1.01–1.03	**0.004**
Positive HIV test	1.39	0.181	1.08–1.79	**0.01**
Number of episodes of UDS*	1.36	0.088	1.20–1.54	**< 0.001**
Number of sexual partners*	1.02	0.012	0.99–1.04	0.16
Alcohol use around sex*	0.84	0.082	0.69–1.02	0.07
Drug use around sex*	0.91	0.265	0.51–1.61	0.74
Never used condoms*	0.88	0.088	0.72–1.07	0.19
Engaged in transactional sex*	0.81	0.083	0.67–0.99	**0.04**
Engaged in sex post-enrollment	1.08	0.115	0.88–1.33	0.48
Used antibiotics in 14 days prior to enrollment	1.14	0.114	0.94–1.39	0.19
STI results				
Negative for NG, CT, TV, MG	1.58	0.214	1.21–2.06	**0.001**
Positive for CT	1.10	0.134	0.86–1.39	0.46
Positive for NG	0.53	0.054	0.44–0.65	**< 0.001**
Positive for MG	1.40	0.225	1.02–1.93	**0.04**
Antibiotic use				
Received CT/ NG antibiotic coverage^µ^	1.14	0.221	0.78–1.66	0.52
Received only CT/NG antibiotic coverage (SOC)^α^	1.12	0.126	0.90–1.40	0.31
Adhered to prescribed antibiotics	0.47	0.233	0.18–1.24	0.13
Received additional treatment post-enrollment	2.67	0.490	1.86–3.82	**< 0.001**

^¥^Confidence Interval; *By self-report in the 6 months prior to study enrollment; ^µ^Received cefixime and doxycycline, also received other antimicrobials; ^α^Received only cefixime and doxycycline

### Multivariable regression models

Table 3 describes the multivariable analysis controlling for potential confounding variables. The significant associations between TTR and additional reported UDS episodes and testing negative for urethral STIs were robust to multivariable analysis; other factors such as a positive MG NAAT were not significant when controlling for other variables. There was a non-significant trend towards increased TTR in those with HIV.

**Table 3:** Multivariable AFT regression models independently fitted for each risk factor of interest

Variable	Failure Time Ratio (95% CI^¥^)	*P* value
Positive HIV test^1^	1.34 (0.89,1.79)	0.09
Number of episodes of UDS*^2^	1.35 (1.17,1.52)	**0.001**
Engaged in sex post-enrollment^3^	1.10 (0.89,1.31)	0.31
Used antibiotics in 14 days prior to enrollment^4^	1.10 (0.90,1.30)	0.30
Negative for NG, CT, TV, MG^5^	1.46 (1.12,1.79)	**0.001**
Positive for MG^6^	1.28 (0.90,1.67)	0.10

1 Covariates include: Age; Number of sexual partners*; Alcohol use around sex*; Never used condoms*; Engaged in transactional sex*; Engaged in sex post -enrollment; Used antibiotics in 14 days prior to enrollment; Negative for NG, CT, TV, MG

2 Covariates include: Number of sexual partners*; Never used condoms*; Engaged in transactional sex*; Engaged in sex post-enrollment; Used antibiotics in 14 days prior to enrollment; Adhered to prescribed antibiotics

3 Covariates include: Number of episodes of UDS*; Never used condoms*; Engaged in transactional sex*; Used antibiotics in 14 days prior to enrollment; Negative for NG, CT, TV, MG; Positive for MG; Adhered to prescribed antibiotics

4 Covariates include: Number of episodes of UDS*; Number of sexual partners*; Never used condoms*; Engaged in transactional sex*; Engaged in sex post-enrollment; Negative for NG, CT, TV, MG; Adhered to prescribed antibiotics

5 Covariates include: Positive HIV test; Number of episodes of UDS*; Engaged in transactional sex*; Engaged in sex post-enrollment; Used antibiotics in 14 days prior to enrollment; Received additional treatment post-enrollment

6 Covariates include: Number of episodes of UDS*; Number of sexual partners*; Never used condoms*; Engaged in sex post-enrollment; Used antibiotics in 14 days prior to enrollment; Adhered to prescribed antibiotics; Received CT/ NG antibiotic coverage

^¥^Confidence Interval; *By self-report in the 6 months prior to study enrollment

## Discussion

In this population of Ugandan men with urethral discharge syndrome and a high prevalence of urethral STIs, nearly 16% did not have symptom resolution 14 days after treatment initiation. Symptom resolution was associated with NG as may be expected from clinical experience and previous studies; >90% of men received anti-gonococcal therapy. In a UK prospective cohort study of 216 patients with gonorrhea using NAATs for diagnosis, 89% men, median TTR was 2 days (IQR 1–3 days) [22]. For the 23% with chlamydia-gonorrhea coinfection, the time to resolution was slightly longer at 3 days [22]. While not a direct measure of TTR of symptoms, in a 2002 study where men and women were treated for gonorrhea, the median time to a negative urine ligase chain rection (LCR) test result in men was 1 day (mean, 1.6 ± 0.14 days) [19]. In this study the median TTR for NG, CT, or both was 4 days (ranges 1–12 days). These data demonstrate that men with UDS in Kampala had a longer time to symptom resolution than in men with experimental gonorrhea or patients with gonorrhea alone or with gonorrhea-chlamydia coinfection.

In our study of Ugandan men with UDS, delayed symptom resolution was associated with previous episodes of UDS in the prior six months and negative tests for four common STI causes of urethritis. This relationship held true when controlling for potential confounders such as possible reinfection following sex post-enrollment, and treatment non-adherence. This may be at odds with other studies where patients who had a treatable STI within the last year had a significantly shorter clearance time than those who did not (means, 1.3 ± 0.08 and 1.9 ± 0.2 days, respectively [*P* = 0.005]) [19]. However, symptom resolution data were not reported in this study.

The mechanism underpinning the relationship between previous UDS episodes and delayed symptom resolution has not been elucidated. Failure of symptoms to resolve may be due to reinfection, persistent inflammation after eradication of infection, treatment failure, or differences in host or pathogen characteristics among those with chronic or recurrent infections. Most men in this study had gonorrhea; gonococcal infection does not produce meaningful mucosal immunity [23]. We hypothesize that men with recurrent UDS had less efficient immune responses to NG (or other urethral STIs) rendering them at greater risk of both recurrent infection and prolonged TTR resolution after appropriate treatment.

In men with HIV there was a non-significant association with TTR; a larger sample size with a greater proportion of men with HIV might reveal a more convincing relationship between HIV and post-treatment STI symptom duration. Alternatively, delayed TTR may be the result of behaviors that increased exposure to different strains of gonorrhea; a UK study demonstrated that the greatest predictor for gonorrhea was a previous history of gonorrhea [OR 4.36 (95% CI 1.78–10.71)] [24]. However, our data demonstrate that NG, independent of a history of UDS, was associated with more rapid TTR and none of the other urethral STIs were associated with reduced TTR. TTR may also be due to other, unmeasured host immunological and pathogen-specific differences, as well as behavioral confounders.

Additionally, almost one-fifth of participants who tested negative for all four pathogens (NG, CT, TV, and MG) demonstrated increased TTR when controlling for potential confounders such as HIV status, recent antibiotic use, treatment non-adherence, and other variables associated with STIs. This observation may be due to symptomatic urethritis caused by infections that were not tested for, such as HSV, Adenovirus, or *Neisseria meningiditis* [4]. Some of these pathogens are not susceptible to the antibiotic regimens administered as part of syndromic management in Uganda or have reduced antibiotic susceptibility [25]. Alternatively, persisting symptoms may be due to additional syndromes such as prostatitis which were not evaluated in this study. Ideally, access to laboratory testing beyond these four pathogens would allow more targeted treatments in the minority of men with persistent symptoms resulting in more personalized therapy and better antimicrobial stewardship.

We explored the potential role of AMR on TTR based on the hypothesis that those more chronically or recurrently impacted by UDS were infected with pathogens that had developed some measure of resistance to the existing antibiotic regimens. Most participants (93.3%) received some combination of an extended spectrum cephalosporin (ESC) and doxycycline or azithromycin [14]. Previous work by our group and others has demonstrated no evidence of reduced sensitivity to ESC in gonococcal isolates from men with UDS in Uganda [14, 15, 26]. These data, combined with the finding that NG positivity was associated with more rapid TTR likely excludes NG AMR as a cause of delayed TTR. In contrast we previously found that 10.7% of men with MG had evidence of macrolide resistance [27]. MG is only eradicated by doxycycline in 30–40% of cases [28], additionally symptom recrudescence may occur after initial resolution following treatment with doxycycline [29].

These locally generated data provide the basis for patient counselling. Knowing and communicating the typical course of a syndrome and the expected time for symptom resolution is crucial for managing patient expectations, providing advice on resumption of sexual activities [4], and reassurance to prevent patients seeking additional antimicrobials. By providing a clear timeline of STI treatment and expected symptom resolution, providers may increase patient adherence to treatment plans. This is critical in ensuring the effectiveness of the treatment, counselling patients when to return if symptoms persist, and preventing the development of AMR by avoiding additional, unnecessary treatment.

### Strengths and limitations

The strengths of this study include a high prevalence of STIs, diagnoses of UDS-associated STIs in a College of American Pathologists-certified laboratory, detailed behavioral data, and high retention for follow up calls. The study has several limitations that warrant consideration. All data on symptoms, symptom resolution, and behaviours such as treatment adherence and sexual activity were self-reported by participants. We used sexual activity post-enrollment as a proxy for reinfection that may not accurately reflect STI transmission. The study duration was relatively short; it is possible that symptom recrudescence occurred after the follow up period. Social desirability and recall biases, or reluctance to disclose sensitive information could affect the reliability of these data. Additionally, a test of cure (TOC) was not performed to verify microbiological eradication. The absence of a TOC introduces uncertainty about the microbiological effectiveness of the treatments administered; symptom or clinical resolution may be a true indicator of successful treatment or symptom amelioration despite persistent infection. ART, CD_4_ counts, or HIV viral loads were not available. Bacterial loads were not assessed, limiting speculation on the relationship between bacterial burden and the duration of symptoms post-therapy. Quantifying bacterial loads in single and multiple urethral STIs may be important for evaluating the dynamics of the infection and determining the effectiveness of interventions in reducing the pathogen burden. Future studies could address these limitations.

There are key public health recommendations that can be generated from this study. Ugandan men with UDS should be counselled on the importance of condom use specifically when symptoms are present, men with a history of UDS may benefit from additional STI prevention interventions. It also calls for surveillance that explores the etiological causes of urethritis beyond typical infections.

## Conclusion

Most men had symptom resolution by day 14. However, over 1 in 6 failed to resolve symptoms in the timeframe expected from experimental and observational studies. There was an association between delayed symptom resolution and previous episodes of UDS, even when controlling for potential confounders. However, the mechanisms behind this association remain unclear, ranging from potential reinfection to persistent inflammation or treatment failure; such observations require further exploration.

## Electronic supplementary material

Below is the link to the electronic supplementary material.

Supplementary Material 1

Supplementary Material 2

## Data Availability

Reasonable requests for deidentified data can be made by contacting the senior author (MMH).

## Abbreviations

AFT: Accelerated failure time
ART: Antiretroviral therapy
CD4: Cluster of differentiation 4
CT: *Chlamydia trachomatis*
HIV: Human immunodeficiency virus
NG: *Neisseria gonorrhoeae*
MG: *Mycoplasma genitalium*
SR: Symptom resolution
TTR: Time to resolution
UDS: Urethral discharge syndrome
TV: *Trichomonas vaginalis*
