# Supplementary material for: Post-treatment time to symptom resolution and associated factors in a cohort of Ugandan men with urethral discharge syndrome
Source: BMC Infect Dis. 2025 Jun 8;25:801. doi: 10.1186/s12879-025-11196-8 (PMC12147313; doi:10.1186/s12879-025-11196-8)
Supplement: Supplementary file 1 — Supplementary Material 1 [file 12879_2025_11196_MOESM1_ESM.docx]

| **1** | **MobiNAAT ID** | [Char, X] | Patient/isolate number |  |
| --- | --- | --- | --- | --- |
| **2** | **EGASP ID** | [Char, X] |  |  |
| **3** | **CLINIC** | [Char, 3] | Sentinel clinic code |  |
| **4** | **DATE** | [Char, 10] | Day/Month/Year of specimen collection | DD/MM/YYYY |
| **5** | **SEX** | [Char, 1] | Sex | 1=male  2=female  9=unknown |
| **6** | **NATIONALITY** | [Char, 1] |  | 1=Ugandan  8=other (please indicate in OTHTRNATION)  9=unknown |
| **7** | **OTHTRNATION** | [Char] |  |  |
| **9** | **TRIBE – if Ugandan** | [Char] |  |  |
| **10** | **RESIDENCE** | DISTRICT | SUBCOUNTY | PARISH |
| **11** | **HIV STATUS** | [Char,1] | This is before today’s visit and before additional testing | 1=positive (go to HIV POSITIVE)  2=negative  9=unknown |
| **12** | **HIV POSITIVE** | [Char, 1] |  | 1=taking ART  2=not taking ART  3=viral load undetectable  4=viral load detectable  9=unknown viral load |
| **13** | **ANAT (MobiNAAT)** | [Char, 1] | Anatomic site of specimen collection | 1=male urethral meatus  2=urine  3=pharynx  4=rectum  5=other  9=unknown |
| **14** | **NUMBER PARTNERS (2)** | [Num,2] | How many sexual partners in previous 2 months | Number:  99=unknown |
| **15** | **NUMBER PARTNERS (6)** | [Num,2] | How many sexual partners in previous 6 months | Number:  99=unknown |
| **16** | **CONDOM USE** | [Num, 2] | Condom use in the last 6 months | 1=always  2=sometimes  3=never  9=unknown |
| **17** | **CONDOM_HIV** | [Num,2] | Condom use with someone of unknown or different HIV status in past 6 months | 1=yes  2=no  9=unknown  12=declined to answer |
| **18** | **NUMBER EPISODES UDS** | [Num,2] | Number of UDS in past 6 months | Number:  99=unknown |
| **19** | **TRANSACTIONAL SEX** | [Num,2] | Gifts, food, shelter, money for sex in last 6 months | 1=yes, given  2=yes, received  3=both given and received  4=no  9=unknown |
| **20** | **AGE** | [Num, 2] | Age in years | Number:  Enter 99= unknown |
| **21** | **SEXOR** | [Char, 1] | History of sex with women, men, or both (all that apply) | 1=women only (heterosexual) (go to SEX_BEHAV_WOMEN)  2=men only (homosexual) (go to SEX_BEHAV_MEN)  3=women and men (bisexual) (go to SEX_BEHAV_WOMEN and SEX_BEHAV_MEN)  9=unknown  12=declined to answer |
| **22** | **SEX_BEHAV_WOM** | [Char] | Sex with women in past 12 months (all that apply) | 1=vaginal with condom  2=vaginal without condom  3=oral with condom  4=oral without condom  5=oral insertive  6=oral receptive  7=oral insertive and receptive  8=anal with condom  9=anal without condom  11=unknown  12=declined to answer |
| **23** | **SEX_BEHAV_MEN** | [Char] | Sex with men in past 12 months (all that apply) | 1=oral with condom  2=oral without condom  3=oral insertive  4=oral receptive  5=oral insertive and receptive  6=anal with condom  7=anal without condom  8=anal insertive  9=anal receptive  10=anal insertive and receptive  11=unknown  12=declined to answer |
| **24** | **ALCOHOL** | [Char] | Alcohol intake in last 6 months | 1=yes (go to ALCOHOL_USE)  2=no  9=unknown |
| **25** | **ALCO_USE** | [Char] | How often do you have a drink containing alcohol? (if 1 go to DRUGS (NOT PRESCRIBED) | 1=never  2=monthly or less  3=2-4 times a month  4=2-3 times a week  5=4 or more times a week  9=unknown  12=declined to answer |
| **26** | **ALCO_DAY** | [Char] | How many standard drinks containing alcohol do you have on a typical day? | 1=1 or 2  2=3 or 4  3=5 or 6  4=7 to 9  5=10 or more  9=unknown  12=declined to answer |
| **27** | **ALCO_6** | [Char] | How often do you have six or more drinks on one occasion? | 1=never  2=less than monthly  3=monthly  4=weekly  5=daily or almost daily |
| **28** | **ALCO_SEX** | [Char] | In the past 6 months have you used alcohol? | 1=before sex  2=and been intoxicated before sex  9=unknown  12=declined to answer |
| **29** | **DRUGS (NOT PRESCRIBED)** | [Char] | Drug use in past 6 months | 1=yes (go to DRUG_TYPE and DRUG_NAME)  2=no  9=unknown  12=declined to answer |
| **30** | **DRUG_TYPE** | [Char] | Type of drug used in past 6 months | 1=smoke  2=snort  3=inhale  4=inject  5=swallow  6=other  7=used before sex  8=intoxicated before sex  9=unknown  12=declined to answer |
| **31** | **DRUG_NAME** | [Text] | All drugs used in past 6 months | 1=  2=  3=  4=  5= |
| **32** | **SYMP** | [Char, 1] | Symptoms of gonorrhea | 1=discharge and/or pain  2=no discharge and no pain  3=burning on urination  4=no burning on urination  9=unknown |
| **33** | **DURATION_SYMP** | [Char] | Duration of symptoms | 1=less than 1 day  2=1day  3=2-5 days  4=6-9days  5=10-14 days  6=more than 14 days |
| **34** | **SEX_SYMPTOMS** | [Char] | Sexually active since developed symptoms | 1=yes with condom  2=yes without condom  3=no  9=unknown  12=declined to answer |
| **35** | **PARTNER** | [Char] | Partner informed of symptoms prior to clinic visit | 1=yes (go to PARTNER_TYPE)  2=no |
| **36** | **PARTNER_TYPE** | [Char] | Which partners informed | 1=all  2=main partner only  3=casual partner only  9=unknown |
| **37** | **ANTIBIOT** | [Char, 1] | Antibiotic use in the past 2 weeks | 1=yes (go to ANTIBIOT_SOURCE)  2=no  9=unknown |
| **38** | **ANTIBIOT_SOURCE** | [Char] | Where were antibiotics obtained from (all that apply) | 1=doctor’s office  2=clinic  3=pharmacy  4=store  5=family or friends  6=bought on street  7=bought on internet  8=other  9=unknown |
| **39** | **NAME OF ANTIBIOT** | [Char] | All that apply | 1=ciprofloxacin  2=doxycycline  3=azithromycin  4=penicillin  5=cefixime  6=ceftriaxone (injection)  7=Cotrimoxazole (Bactrim)  8=other  9=unknown |
| **40** | **DURATION OF ANTIBIOT** | [Char, days] |  | Number:  9=unknown |
| **41** | **PARTNER NOTIFICATION** | [Char] | Are you planning to inform sex partners of probable STD diagnosis | 1=yes, all  2=yes, main only  3=yes, casual only  4=no  9=unknown  12=declined to answer |
| **42** | **STD AWARNESS** | [Char] | Awareness of following conditions (list all) | 1=chlamydia  2=gonorrhea  3=trichomonas vaginalis  4=mycoplasma genitalium  5=syphilis  6=HIV  7=Hepatitis B  8=herpes |
| **43** | **STD_TRANS** | [Char] | If yes to STD AWARNESS. Ask if the infection can be passed through sex. E.g. 1/1, 2/2 | 1=yes  2=no  9=unknown |
| **44** | **STD_SYMP** | [Char] | If yes to STD TRANS. Ask if selected infection has symptoms or not. E.g. 3/1, 5/9 | 1=yes  2=no  9=unknown |
| **45** | **TRMT1** | [Char, 1] | Primary treatment for gonorrhea (given by clinician) | 0=none  1= cefixime 400mg  2=ceftriaxone 250 mg  3=ceftriaxone 500mg  4=azithromycin 2 gm  8=other (please indicate in Other Treatment 1, OTHTRTMT1)  9=unknown  11=medication given directly to patient  12=prescription given to patient to get medication elsewhere |
| **46** | **OTHTRMT1** | [Char, 15] | Other treatment not listed as code for TRMT1 (given by clinician) | If code “8” was entered for Treatment 1, please type in the name and dosage of the drug used for primary treatment of gonorrhea.  11=medication given directly to patient  12=prescription given to patient to get medication elsewhere |
| **47** | **TRMT2** | [Char, 1] | Second antibiotic used as part of dual therapy for gonorrhea (and treatment of chlamydia or non-gonococcal urethritis) (given by clinician) | 0=none  1= doxycycline/tetracycline  2= azithromycin 1 gm  3=azithromycin 2 gm  8=other (please indicate in OTHTRMT2)  9=unknown  11=medication given directly to patient  12=prescription given to patient to get medication elsewhere |
| **48** | **OTHTRMT2** | [Char, 15] | Other treatment not listed as code for TRMT2 (given by clinician) | If code “8” was entered for TRMT2, please type in the name and dosage of the drug used for primary treatment of gonorrhea.  11=medication given directly to patient  12=prescription given to patient to get medication elsewhere |
