# Supplementary material for: Post-treatment time to symptom resolution and associated factors in a cohort of Ugandan men with urethral discharge syndrome
Source: BMC Infect Dis. 2025 Jun 8;25:801. doi: 10.1186/s12879-025-11196-8 (PMC12147313; doi:10.1186/s12879-025-11196-8)
Supplement: Supplementary file 2 — Supplementary Material 2 [file 12879_2025_11196_MOESM2_ESM.docx]

ID number:

Date of call:

Study visit date:

Visit code

Day 7

Day 14

Day 21

Study site number:

RA’s initials

Attempt number 2

Date:

Time:

Outcome:

1. Spoke to participant

2. Phone rings with No answer

3. Somebody else answered the phone

4. Uncontactable

5. Other

4. Other

Attempt number 1

Date:

Time:

Outcome:

1. Spoke to participant

2. Phone rings with No answer

3. Somebody else answered the phone

4. Uncontactable

5. Other

4. Other

Attempt number 4

Date:

Time:

Outcome:

1. Spoke to participant

2. Phone rings with No answer

3. Somebody else answered the phone

4. Uncontactable

5. Other

4. Other

Attempt number 3

Date:

Time:

Outcome:

1. Spoke to participant

2. Phone rings with No answer

3. Somebody else answered the phone

4. Uncontactable

5. Other

4. Other

Verification of participant: Ask for study code or study ID or prior agreed passcode

Hello, my name is ______________, I work at IDI and am calling to check how you are after you recently visited the clinic and took part in the study looking at sexually transmitted infections. As we discussed on the day I have a few short, simple questions to ask you. Can I proceed now?

Yes: ­­­­­­­­­­­­­­­­­­­_____________________________________ (PROCEED TO **PART A**)

No: When can I call you back, what day and time? ­­­­­­­­­­­­­­­­­­­­­____________________________________________

**PART A:**

1. Did you take the medication as prescribed by the clinic? Y/N (If Y go to part B, if N go to 2)

2. If not why not? ☐ Too expensive, ☐ not able to get time to go to pharmacy, ☐ symptoms improved, ☐ received alternative treatment, ☐ did not think that the medicine would work

**PART B:**

1. Have your symptoms gone away completely? Y/N (If Y go to 2, If N go to 3)

2. How many days after antibiotic treatment did the symptoms go away? (go to 5)

3. Have your symptoms improved? On a scale of 0 to 10 (0 shows not at all and 10 fully improved) Y/N

4. How are your symptoms today compared to your last visit? (Visit means either clinic visit or telephone follow up): worse, the same,  slightly better,  much better

5. Do you have discharge from the penis? Y/N

6. Do you have burning when you urinate? Y/N

7. Have you been back to the clinic since your last visit? Y/N (If Y go to 8, if N go to 11)

8. How many days ago was this?

9. Did they give you any more treatment? Y/N (If Y go to 10)

10. Please lists the medications:

11. Did you go to ANY facility for treatment since your last visit (included pharmacy, medical doctor, store,  street vendor etc.) Y/N (if Y go to 12, if N go to **Part C**)

12. What type of person did you see (e.g. pharmacist,medical doctor traditional healer, ☐ other please state):

13. What treatment did they give you?:

14. Was it tablets/pills, injection, cream, herbs, ☐ other (please state)?:

15. How many days did you take it for?

**PART C:**

1. Have you had sex since your last clinic visit or our last follow up phone call? Y/N (if Y go to 2, if N go to **Part D**)

2. Did you use a condom during your last sexual encounter? Y/N

3. How many sexual partners have you had since then?:

4. How many of these are NEW partners (i.e. first time you had sex since last visit)?:

5. Were the partners Male, Female, Both, prefer not to answer?

**PART D:**

1. Since your last visit did you inform ANY of your partners that you were treated for STI? Y/N (If Y go to 2, If N go to 5)

2. How many in TOTAL did you inform?

3. Did you have sex with any that you informed? Y/N (if Y go to 4)

| Partner number | Main partner Y/N | Casual Partner Y/N |
| --- | --- | --- |
| Partner 1 |  |  |
| Partner 2 |  |  |
| Partner 3 |  |  |
| Partner 4 |  |  |
| Partner 5 |  |  |
| Partner 6 |  |  |

4. Did you tell them about the STI before or after you had sex with them?

| Partner number | Before sex | After sex |
| --- | --- | --- |
| Partner 1 |  |  |
| Partner 2 |  |  |
| Partner 3 |  |  |
| Partner 4 |  |  |
| Partner 5 |  |  |
| Partner 6 |  |  |

5. Do you plan to tell them? Y/N (If Y go to 6, if N go to 7)

6. How will you tell them?: Face-to-face, telephone, email, SMS, other (list)

7. Why do you think you have not told them?: Not seen them, not sure what to say, worried that they might tell someone else, embarrassed, other (list)

8. Is there any way that would make it easier to communicate this information to your sexual partner(s)? Y/N (if Y go to 9, if N go to **Thank you**)

9. How? Clinic inform them,  peer worker inform them, other (list)

12. How would you rather they be informed?: SMS, phone call, email, other (list)

Thank you for your time today. Your information will be kept private and confidential.

Do you have any questions? Y/N

If Yes, list:

If answered list responses:

We will call you again in 7 days.

If this is day 21 visit say, “thank you for taking part in the study, we appreciate your assistance.”
